# A reconfigurable NAND/NOR genetic logic gate

**DOI:** 10.1186/1752-0509-6-126

**Published:** 2012-09-18

**Authors:** Angel Goñi-Moreno, Martyn Amos

**Affiliations:** 1 School of Computing, Mathematics and Digital Technology, Manchester Metropolitan University, Manchester M1 5GD, United Kingdom

**Keywords:** Synthetic biology; Boolean logic; Multifunctionality

## Abstract

**Background:**

Engineering genetic Boolean logic circuits is a major research theme of synthetic biology. By altering or introducing connections between genetic components, novel regulatory networks are built in order to mimic the behaviour of electronic devices such as logic gates. While electronics is a highly standardized science, genetic logic is still in its infancy, with few agreed standards. In this paper we focus on the *interpretation* of logical values in terms of molecular concentrations.

**Results:**

We describe the results of computational investigations of a novel circuit that is able to trigger specific differential responses depending on the *input standard* used. The circuit can therefore be *dynamically reconfigured* (without modification) to serve as both a NAND/NOR logic gate. This multi-functional behaviour is achieved by a) varying the *meanings* of inputs, and b) using *branch predictions* (as in computer science) to display a constrained output. A thorough computational study is performed, which provides valuable insights for the future laboratory validation. The simulations focus on both single-cell and population behaviours. The latter give particular insights into the spatial behaviour of our engineered cells on a surface with a non-homogeneous distribution of inputs.

**Conclusions:**

We present a dynamically-reconfigurable NAND/NOR genetic logic circuit that can be switched between modes of operation via a simple shift in input signal concentration. The circuit addresses important issues in genetic logic that will have significance for more complex synthetic biology applications.

## Background

The emerging field of synthetic biology [1-7] applies rational engineering principles to the (re)design of biological systems. Work in this area has often focussed on the creation of small-scale genetic devices, such as oscillators [8], toggle switches [9,10], clocks [11], Boolean logic gates [12-15] and half-adders/subtractors [16].

One interesting aspect of such devices concerns their potential for *multifunctionality* (that is, the possibility that devices may switch between different operating modes, depending on some external signal). Most existing engineered gene circuits have been constructed to perform a *single* function, but recent results suggest that such devices may be able to implement *multiple* functions [11,17]. This property is often observed in neuronal networks [18], as it allows organisms to select multiple behavioural “programs” using the same group of neurons. The ability to engineer multifunctionality into genetic circuits may have significant performance benefits when a *range* of different responses or behaviours is required. In this paper we describe a model for such a genetic circuit, which may be dynamically reconfigured (without modification) to serve as both a NOR gate (output “1” only when both inputs absent) and a NAND gate (output “0” only when both inputs present), depending on its input. We give the results of single cell computational experiments, before showing how two-dimensional, population-based simulations can shed valuable light on both the behaviour of the system and its beneficial features.

We describe this circuit in the context of our previous work [19] on *continuous computation* in engineered gene circuits. By “continuous computation”, we mean gene-based computation that maximises the period during which outputs are valid and “readable”, by using “real-valued” signals.This addresses issues of reliability in such circuits, by (a) carefully interpreting binary signal values in terms of continuous/analogue value *thresholds* over time, and (b) using the concept of *branch prediction* (taken from computer architecture). During the execution of a program, a “fork” may occur as the result of a conditional statement (e.g., “if X is true, then do A, else do B”), as in the operation of a logic gate, where the output depends on the inputs. Branch prediction is a technique generally used for saving time when a device faces this kind of decision, and a prediction may be either *conditional* or *unconditional*. The latter (studied in [19]) is used when the probability of one branch being taken is significantly higher than the other; in this case, the high probability branch is taken by *default*, and the situation is only corrected if it transpires that the decision is incorrect, based on the expression evaluation. The circuit proposed here uses *conditional* prediction, by assuming that the previous output expressed will be carried forward to the *next output* (and correcting itself if this is not the case) before processing the inputs.

**Figure 1:**
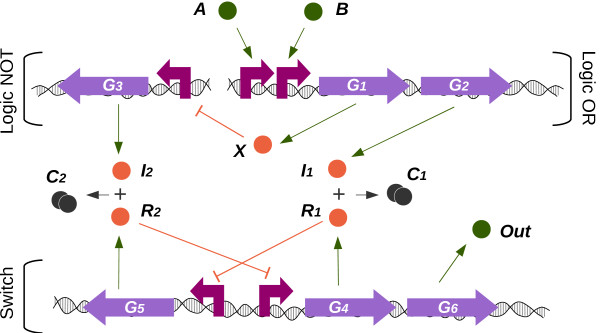
**Proposed genetic circuit.** Our circuit is composed of three well differentiated parts: 1) The OR function, with inputs *A* and *B*, inducing the expression of *X* and *I*_1_ by binding to their correspondent promoter; 2) The NOT function, with output *I*_2_, controlled by a constitutive promoter which is repressed by *X*; 3) A switch, made up of two constitutive promoters which express repressors *R*_1_ and *R*_2_ as well as the reporter *Out*. Protein complexes *C*_*i*_ are formed by the sequestration of the *R*_*i*_ by *I*_*i*_.

We present our circuit design in Figure 1. Although it can behave as either a NAND or a NOR gate, for clarity we describe here only the NOR logic interpretation of the circuit, and present the multi-functional behaviour in the Results and discussion section. The NOR (negated OR) logic circuit is formed by three sub-components: 1) a logic OR gate, 2) a logic NOT gate (or inverter) and 3) a genetic switch. As the NOR logic function is an inverted OR, the output of the inverter (*I*_2_) could be taken as the output, along the lines of a *classical* genetic NOR [20]. However, in our design, the output is denoted by the protein expressed by the switch, (*Out*), as this allows us to implement branch prediction (that is, changing the switch means that our branch prediction needs to be corrected). In the Results and discussion section we highlight the advantages of the proposed circuit.

The inputs of the circuit, represented by molecules *A* and *B*, induce the expression of both genes *G*_1_ and *G*_2_(by binding to their correspondent upstream promoters), which produce, in turn, proteins *X* and *I*_1_(*Inducer*_1_) respectively. Product *X* represses the production of *I*_2_(*Inducer*_2_), which is expressed by the inverter using gene *G*_3_. Inducers *I*_1_and *I*_2_are in charge of controlling the switch, and change its direction. This third part of the circuit (the switch) is formed by two promoters which control the expression of three genes. Repressor *R*_1_ represses the expression of gene *G*_5_ unless inducer *I*_1_ sequesters it, forming the complex *C*_1_ (which has no functionality in the circuit). Symmetrically, *R*_2_ represses the expression of both genes *G*_4_and *G*_6_which, in turn, produces the reporter *Out*.

We now briefly consider the possible implementation of our system. The two main components we use are a genetic toggle switch and NOR gate, both of which have previously been successfully demonstrated in the laboratory [9,20]. The main novelty in our proposed scheme (in terms of its implementation) lies in the *connection* between both components. We believe that this is where attention should be focussed during future laboratory work. The outputs of the NOR gate (that is, the *inducers* of the switch) are inhibitors of the *R*_1_ and *R*_2_repressors. By being sequestered, the repressors are rendered inactive, and the implementation of such a scheme is supported by a recent study [21], in which examples of such negative inhibition are demonstrated. Further investigations may also focus on alternative implementations of the connection scheme, without altering the fundamental behaviour of the device. One possible route to this may lie in *directly* repressing the switch promoters, instead of implementing the protein-protein interaction. There is also the possibility of using RNA-based logic to implement connections, as described recently in [22]. The key consideration that should inform the engineering process is the fact that the *maximum* expression level of the NOR output must be at least equal to the maximum expression level of the switch inputs. This is what allows the switch to “flip”. Conversely, the *minimum* expression level of either NOR output must be lower than the minimum levels of either switch input. These two features allow the device to have the desired multi-functional behaviour.

In terms of traditional electronic logic, our circuit therefore corresponds to a system that produces, by default, a “high”, or “1”, signal in the absence of *any* input signals equal to “1”. As soon as *either* inputs equal “1”, the output signal is “pulled low” to “0” (classical NOR behaviour). Once all “1”-valued inputs are removed, the circuit defaults back to “high”. In the Results and discussion section, we study the dynamics of the circuit and - more importantly - the *meaning* of a logic “1”-valued input, from which the multi-behaviour feature of the system is derived.

## Results and discussion

We perform a number of computational simulations (model details are specified in the Methods section), with the two main aims of investigating the behaviour of the branch-predicting NOR gate, and then examining its potential as a reconfigurable device. In both cases, we perform single-cell and population-based experiments, to investigate both the internal dynamics of the circuit and its effect on a spatially-distributed collection of cells (which might be used in a realistic synthetic biology application).

### Single cell NOR

We first emphasise the difference between *static* and *dynamic* (i.e., continuous) observations. Static measurements are performed by testing a single logic case (input setup), observing from the initial state of the circuit until a steady-state is reached. Dynamic measurements are taken once initialisation has occurred, and the logic input cases are modified sequentially. The outputs obtained are not always consistent, and we conclude that the continuous paradigm is more appropriate (i.e., robust) for these circuits.

**Figure 2:**
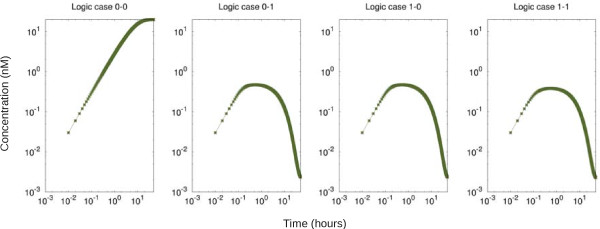
**Static observations of circuit.** Four logic cases (combination of two *binary* inputs) tested with logic “0” fixed at 0 nM and logic “1” at 5 nM (deterministic simulation). Perfect NOR behaviour is observed, as the output *Out* is only expressed at a high level for the input case 0-0. In the other cases, *Out* expression is repressed (initially expressed slightly due to initial *I*_2_ concentrations). Axes shown in logarithmic scale for both Time (hours) and Concentration (nM).

Figure 2 shows *static* observations of the NOR gate. We represent its output value in terms of the concentration of *Out*: 0 nM corresponds to an input value of “0”, and 5nM corresponds to an input value of “1”. As expected, we only observe an high positive output when both inputs are absent (i.e. zero). In the other cases, although the output is initially expressed due to the constitutively expressed inducer *I*_2_, it is soon repressed due to input action.

It is important to note, however, that this performance is only observed when the system starts from a “pristine” (i.e. unused) state. Therefore, this behaviour is only useful if the circuit is intended for “single use”. In non-trivial synthetic biology applications, it may well be the case that a circuit is used many times, with different inputs, so it is important to test its behaviour over an extended period. Once the system has been initialised with a set of inputs, we therefore need to switch over to a dynamic observation model [19].

**Figure 3:**
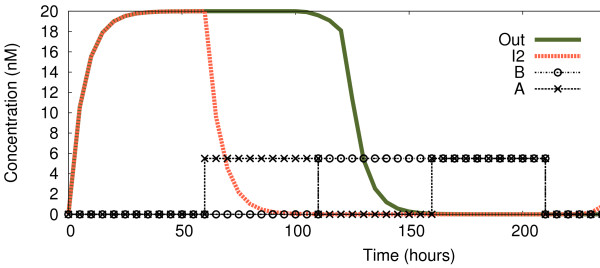
**Continuous observations of circuit.** Deterministic study of the change of *Out* and *I*_2_ over time, while the four logic cases are introduced dynamically. Until *t* ≈ 60 both inputs are “0” (case 0-0); from 60 until *t* ≈ 110 input *A* is a logic “1” (case 1-0); until *t* ≈ 160 input *A* is “0” while input *B* is “1” (case 0-1); until *t* ≈ 210 both inputs are 1; from there onwards both inputs come back to “0”. Logic “0” represented by 0 nM, logic “1” by 5 nM.

In order to study the behaviour of the logic gate over time, we compare the concentrations of inducer *I*_2_(which would be a “traditional” NOR output) and our output signal, *Out*. Figure 3 shows the concentration of these two proteins over time while the inputs to the circuit are changed dynamically. We observe correct branch prediction, in that an output *tends* to reflect the previous output. Both *I*_2_ and *Out* are produced at the outset, when there are no inputs to the system. As soon as one of the inputs is introduced (*A*, giving an input of 1 after *t*≈60 hours), the correct output must be “0”. A NOR without prediction, represented here by *I*_2_, would switch off the expression almost immediately, but *Out* is still expressed for some time (that is, there is a delay in pulling the output signal low, which starts to occur just at the end of the 1-0 input period, and continues through the subsequent 0-1 input period). This is due to the fact that it takes some additional time to “flip” the switch that is controlling *Out*, but this delay makes our circuit much more reliable (as we shall see when considering noise). By illustration, unwanted noise in the input will instantly affect *I*_2_, but the noise needs to be very persistent in order to affect *Out*. The same behaviour is observed when the proteins are again expressed (*t*≈210 in Figure 3), where *Outdelays* its expression before returning to “1” (since both inputs return to “0”). In both delays, the system is still *predicting* the previous output.

### Single cell NOR with noise

**Figure 4:**
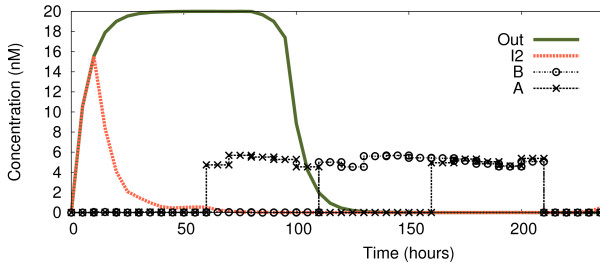
**Continuous observations of circuit, with added noise.** Change of *Out* and *I*_2_ over time while the four logic cases are not homogeneous due to noise in input signals (stochastic inputs). During the case 0-0 (until *t* ≈ 60) the logic value “0” varies within the range [0…0.05] nM; for the case 1-0 (until *t* ≈ 110) input A varies within the interval [4.5…5.5] (logic “1”) while input B still varies within the previous interval for a logic “0”; same variation ranges for “0” and “1” during cases 0-1 (until *t* ≈ 160) and 1-1 (until *t* ≈ 210); From there, again the case 0-0 but with another definition of logic “0”, varying within the range [0…0.005].

The effect of noisy inputs is shown in Figure 4, where both input concentrations are affected by stochastic noise within different intervals. We highlight in this way the different behaviour of the *classic* NOR represented by the product *I*_2_and our approach represented by *Out*. During the 0-0 input phase (until *t*≈60) the input values vary within the range [0…0.05] nM. This underlines the importance of interpreting binary values in terms of *ranges* of analogue biological variables. The small changes in *A* and *B* test the definition of logic “0” in this experiment. Higher concentrations within that range are enough to stop the production of the inducer *I*_2_ (there is always a small concentration during this time), but are still understood as a logic “0” by our circuit (thus *Out* is constantly expressed). That “understanding” is precisely due to the engineered predictive behaviour: as the *previous* state was 0-0 (i.e., the initial situation), the circuit keeps that state regardless of the noise present.

Dynamic predictions are observed after the 0-0 case, as the initial - *static* - conditions are no longer valid. During the 1-0 input phase (until *t*≈110) input *A* varies within the interval [4.5…5.5] nM (logic “1”) while input B is still varying within the range [0…0.05] nM (logic “0”). In this scenario, the expression of protein *I*_2_is completely repressed, as the circuit senses this input as a clear logic “1” -not noise- for both *I*_2_and *Out*. The same thing happens during the next (0-1 and 1-1) cases, where the inputs vary within the same intervals for both logic values and output concentrations are the same. In order to get a more valuable insight into the system, we change the meaning for a logic “0” for the final case 0-0 (from *t*≈210) where it varies within the interval [0…0.005]. Such a low signal causes the production of *I*_2_but not at *full* capacity due to existing *X* repressors in the system. That amount, which can be interpreted as a positive output of *I*_2_, it is not enough to change the direction of the switch. Thus, the low inputs (A and B) are interpreted as *noise* for our system and *Out* will stay in the previous state, which is a “0” output. The noise causes an unclear signal to be received at the input: neither a clear logic 0 nor a clear logic 1, thus the system predicts the previous behaviour.

**Figure 5:**
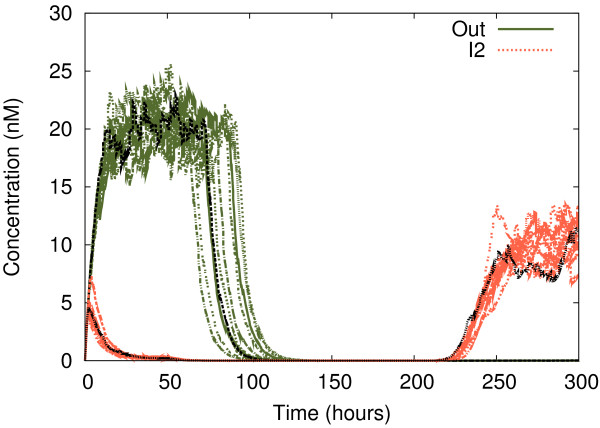
**Full stochastic simulation with noise.** Stochastic simulation (eleven runs) of the changing behaviour of *Out* and *I*_2_. All input values and times are taken from Figure 4. All expression products in the system are subject to randomness,with Gaussian noise applied to all concentrations in the integration steps of the equations.

In Figure 5, the results of a full stochastic simulation are shown, using the input profile of Figure 4. The objective is to test the system in a situation where the concentrations of *all* proteins are subject to randomness. For this purpose, we added Gaussian noise (*mean* = value, *standard deviation* = value · noise) at every iteration of the integration. As we see, the overall behaviour remains the same, which allows us to conclude that the *logical input values* are the key factor determining the correct functioning of the genetic gate. We also observe that the levels of *Out* are more distinct (in terms of their mapping onto binary values) than the levels of *I*_2_.

### Population-based NOR

**Figure 6:**
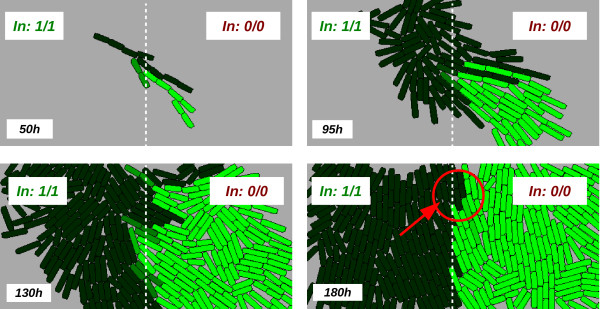
**Population-based simulation.** Sequential observation of a simulated growing population. The surface on which the cells are growing in contains the inputs with both inputs present in the left half (input logic “1”, established at 1.5 nM for this simulation, as before) and neither input present in the right half (input logic “0”, fixed at 0.0 nM, as before). The output *Out* is represented as if it were a green fluorescent protein: high expression corresponds to a bright green colour of the cells. The high mobility of cells after 50 and 95 hours (due to there being plenty of free space available) lets us see the graphical pattern produced by the predictive behaviour of the circuit. When the population is very crowded (after 180 hours) the behaviour of the circuit is directly proportional to the surface features. Red circled region: wrong predictions being *resolved* by changing the direction of the switch. Generation time of cells = 12h.

We now study the behaviour of the circuit inside a population of simulated cells growing on a two-dimensional surface. We use an agent-based simulation approach, which considers the physical factors within the system (cell-cell pressure, collisions, movement, etc.) The first 2-dimensional experiment considers the surface divided in two different areas depending on the inputs they contain (amounts of A and B) as seen in Figure 6. The left-hand side of the surface has both input molecules present (1-1), and the right-hand side has no input molecules present (0-0). As before, the logic “1” concentration is set at 5nM and the logic “0” is set at 0nM. We begin with a single cell in the centre of the surface; cells are “washed out” at the edges, and we assume the constant presence of nutrients (as in a chemostat). In these simulations, we assume *Out* to be a green fluorescent protein (for visualisation purposes), and the cell generation time is kept very high (around 12 hours), in order to aid visualisation. That is, because of the delay caused by the switch, we would not be able to observe the desired behaviour at this scale with much lower cell doubling times. Thus, in order to perform the spatial study with a scale that allow us to visualise single cell shapes we increase the doubling time to 12 hours (more details in the Methods section).

We depict the behaviour of the simulated colony in Figure 6, starting with a single cell in the centre of the region. As the number of cells increases, those to the right-hand side eventually exhibit fluorescence, as they inhabit the 0-0 region, while those to the left (in the 1-1 region) show no fluorescence, as expected.

After 50 hours, we notice some cells on the right-hand side that are not producing light, when they should actually display a high output concentration. This is due to the fact that those cells are *moved* from the left-hand side (1-1 case) at high speed while they are being pushed strongly. Therefore, the circuit inside those cells has not had time enough to respond and start expressing *Out* (we recall the gap of Figure 3). After 130h the cells clearly signal the input concentration corresponding to the inputs on the surface (any single-cell “errors” are due to cell movement and/or stochasticity).

**Figure 7:**
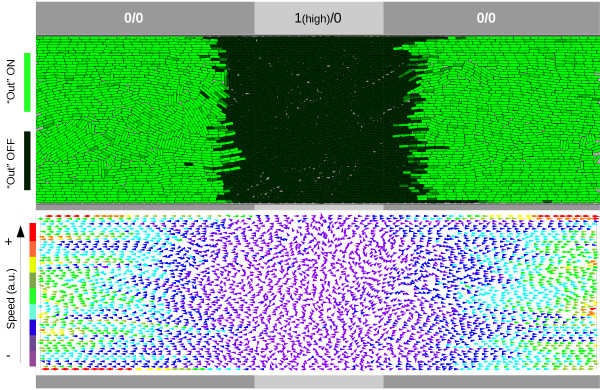
**Effect of cell movement on accuracy.** Spatial delay in response due to time spent in *changing* the direction of the switch. The population is growing from the centre of the longitudinal trap (cells washed out at edges) and the image is taken after 300 hours. The middle sector of the trap (light gray) has only one input (case 1/0) at a high level (4.5nM, which leads to a NOR function), and the remaining area (dark gray) has no input (logic case 0/0). The velocity vector field (lower image) shows the direction and magnitude (colour scheme) of the speeds of every cell at the same time (300 hours).

We now look at the issue of cell movement in more detail. In Figure 6 we show a population growing in a *half and half * world (in terms of input signal distribution), until we obtain an almost perfect pattern (the outputs matched the inputs, spatially speaking). This precision is obtained due to the low speed (and null direction) of the cells in the centre. In Figure 7 we show the result of a subsequent experiment to investigate the effect on pattern formation of a higher velocity field.

In this experiment, the environment (a longitudinal trap) is divided into two zones: the *centre*: with a 1/0 input profile, and the remaining area, with a 0/0 input profile. The cells start growing once inoculated at the centre of the trap (about 100 cells are placed at the beginning). Obviously, as seen in the velocity field (bottom of Figure 7), the cells move in one of two different directions, depending on their physical location: from centre to left, and from centre to right (due to pushing forces while growing). When the cells reach the 0/0 area we would expect a cell’s circuit to display a “1” state. This is what we observe, but with a time delay, as seen in the previous differential study. In this particular case, the higher a cell’s velocity, the more space it will cover before processing the inputs. This explains the gap between the beginning of the 0/0 area and the region in which the cells start expressing the output *Out*. This time-space delay plays a very important role in attempts to generate *specific patterns* in a cell population. If that is the case, there is a key parameter to bear in mind: the *velocity* of the cells, which can - of course - vary within the same colony (as in Figure 7).

It is important to notice that our circuit offers significant possibilities for pattern formation or sensing studies. Instead of recognising only a logic “1” and a logic “0”, the circuit is also able to distinguish between a *high* logic “1” and a *low* logic “1”, and change its behaviour accordingly. We now investigate further this useful property.

### Multi-functional behaviour

In this Section we use different sets of simulations to illustrate one of the main features of the circuit: the possibility of reusing it (*without modification*) for evaluating a function other than NOR. The key factor lies in how we define input “1”. In contrast to our previous experiments, where this is denoted by an input concentration of 5nM, here we reduce the input “1” concentration to 1.5nM. By “flipping” the high input signal from 5 to 1.5nM, we obtain a change in functionality, from NOR to NAND (negated AND). Such a possibility could prove invaluable in terms of saving space in a hybrid bio-device, if differential behaviour is required for a range of input values. We compare our approach to that of Budyka [23]; their gates use *light* as an input, and the functionality of a gate may be altered by changing its wavelength (see also [24], in which the behaviour of a promoter is flipped between that of an amplifier and an OR gate using different inducer concentrations).

**Figure 8:**
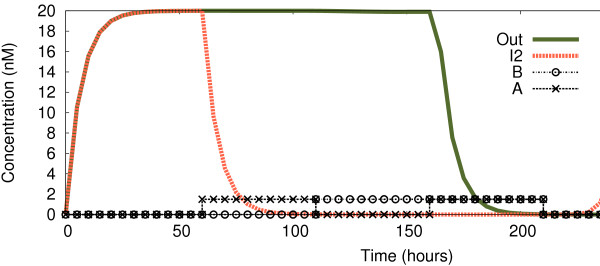
**Continuous observations of circuit with lower concentration for logic “1”.** Change of *Out* and *I*_2_ over time while the four logic cases are being introduced dynamically. Until *t* ≈ 60 both inputs are “0” (case 0-0); from there until *t* ≈ 110 input *A* is a logic “1” (case 1-0); until *t* ≈ 160 input *A* is “0” while input *B* is “1” (case 0-1); until *t* ≈ 210 both inputs are “1”; from there onwards both inputs come back to “0”. As before, logic “0” represented by 0 nM, but this time logic “1” is represented by 1.5 nM.

In Figure 8 we show the behaviour of the circuit with a concentration of 1.5nM representing input logic “1”, in contrast to the 5 nM of Figure 3. We observe how *I*_2_reacts to the changes in exactly the same way as before, thus displaying a NOR behaviour. However, the *Out* signal now gives the correct output reading for a NAND logic function (which returns 0 if and only if both inputs are 1). When *both* inputs are introduced (*t*≈160), the circuit stops producing *Out*, and does not express it again until the inputs are gone (*t*≈210 plus the time needed for the degradation of *R*_2_).

**Figure 9:**
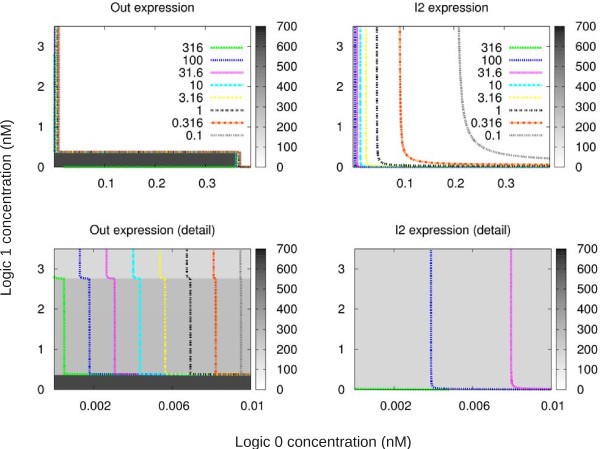
**Effect of different input concentration values.** Surface graphs that explore the behaviour of the circuit for different logic “1” and “0” concentrations. For each pair of logic “0” (*x* axis) and logic “1” (*y* axis) the experiments shown in Figures 3 and 8 are performed, and the cumulative values of *Out* and *I*_2_ over time are recorded. Those values are depicted in two ways: (1) colour surface (greyscale) with a linear scale from 0 to 700 (low precision as mean values are shown for intervals), and (2) contour lines (colour) with a logarithmic scale for detail behaviour. Output values (surface) shown in arbitrary units corresponding to the cumulative value.

Figure 9 shows the behaviour of *Out* and *I*_2_over time when *different* input concentrations are used as logic values. We show logic “0” on the *x*-axis of the surface graphs, and logic “1” on the *y*-axis. The *z* axis (surface view) represents the cumulative value of the targeted output protein (*Out* or *I*_2_depending on the graph) over 300 simulated hours, while inputs are changed according to the profile of Figures 3 and 8. For example, if we fix the value of logic “0” to 0 nM, we observe a change in the concentration of *Out* when the concentration of logic “1” exceeds 2.8 nM, when *Out* abandons the contour line of 316 and enters the area of 100 (which means it has been expressed for less time during the 300 hours). However, that change is *not* present in the expression of *I*_2_, where the scenario is more homogeneous. This feature is the root cause of the multi- functional behaviour we have just demonstrated.

**Figure 10:**
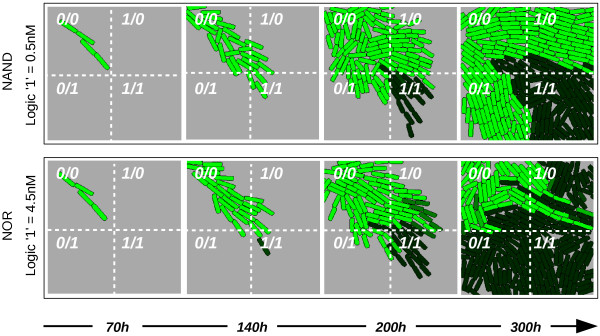
**Multi-functional behaviour of the circuit.** Spatial cell growth simulation shown, where the inputs are *embedded* in the surface as follows: top-left quadrant has no inputs, top-right quadrant has input *A* (1-0), bottom-left has only input *B* (0-1) and bottom-right has both inputs *A* and *B* (1-1). Both rows show the expression of *Out* over time, but for different logic “1” *standards*: 4.5 nM for a NOR logic function (top) and 0.5 nM for a NAND logic gate (bottom). Logic “0” is 0.0 nM in all simulations. a.u.: arbitrary units. Generation time of cells = 24h.

Multi-functional behaviour in cell populations is shown in Figure 10, where a bacterial colony grows on a surface with inputs that are spatially distributed as follows: top-left quadrant has no inputs (0-0), top-right quadrant has input *A* (1-0), bottom-left has input *B* (0-1), and bottom-right has both inputs, *A* and *B* (1-1). The top row shows the level of *Out* when logic “1” is fixed to 0.5 nM, and the bottom row shows it set to 4.5 nM (in both cases logic “0” is set to 0 nM). We clearly observe the difference between the NOR and NAND behaviour of the same circuit placed in different input scenarios.

## Conclusions

The definition of logical values is of vital importance if different synthetic regulatory networks are intended to work together. Here we show that a given genetic circuit can display very different behaviours, depending on the thresholds of a specific input logic signal. This will be of significance for future genetic circuit design. The circuit proposed in this paper harnesses this *fuzzy* behaviour by reconfiguring its behaviour between the NAND and NOR logical functions in response to different input standards. In this way, the circuit can be reused for either of those two functionalities without modification. We highlight the importance of computational studies in order to find abnormal behaviours inside circuits, and to identify the key features of a system. Although in this work we take all parameter values from the literature, the simulation results help us to focus on the specific areas of interest to parts selection for future laboratory validation.

## Methods

Due to the large *size* of the circuit - with 5 promoters, 10 protein species and 6 genes - the mathematical model is reduced to 6 Michaelis-Menten equations (4 to 8). However, a full deterministic model of the first expression product (*X*) is built in order to: (1) check if the approximation is correct, and (2) make a good setup of the parameters in equations.

Twelve biochemical reactions describe the expression of *X*, where both inputs *A* and *B* are involved as well as the gene *G*_1_. These reactions are:

(1) $$\begin{array}{c} A+G_{1}\overset{k_{1}}{\underset{k_{-1}}{⇌}}G_{1}^{a}\\ B+G_{1}\overset{k_{2}}{\underset{k_{-2}}{⇌}}G_{1}^{b}\\ B+G_{1}^{a}\overset{k_{3}}{\underset{k_{-3}}{⇌}}G_{1}^{\mathrm{ab}}\\ A+G_{1}^{b}\overset{k_{4}}{\underset{k_{-4}}{⇌}}G_{1}^{\mathrm{ab}} \end{array}\;\begin{array}{c} G_{1}^{a}\overset{k5}{\to }G_{1}^{a}+X\\ G_{1}^{b}\overset{k6}{\to }G_{1}^{b}+X\\ G_{1}^{\mathrm{ab}}\overset{k7}{\to }G_{1}^{\mathrm{ab}}+X\\ X\overset{k8}{\to }\phi \end{array}\;\begin{array}{c} A\overset{k9}{\to }\phi \\ B\overset{k10}{\to }\phi \\ \phi \overset{k11}{\to }A\\ \phi \overset{k12}{\to }B \end{array}$$

where $G_{1}^{a}$ denotes the gene with input *A* bound to its corresponding promoter, $G_{1}^{b}$ is the gene with input *B* bound to the other promoter and $G_{1}^{\mathrm{ab}}$ represents the gene with both inputs bound. Regarding the rates: *k*_1_ and *k*_2_are the binding rates of A and B, respectively, to their promoters when *G*_1_ has no protein bound; *k*_−1_ and *k*−2 are the unbinding rates of the previous reactions; *k*_3_and *k*_4_ are the binding rates of B and A to $G_{1}^{a}$ and $G_{1}^{b}$, respectively; *k*_−3_ and *k*−4 denote the unbinding rates of the previous reactions; *k*_5_, *k*_6_ and *k*_7_ are the active transcription rates of *X* by $G_{1}^{a}$, $G_{1}^{b}$ and $G_{1}^{\mathrm{ab}}$ respectively; *k*_8_, *k*_9_ and *k*_10_ are the degradation rates of *X*, *A* and *B*; and *k*_11_and *k*_12_ are the creation rates of inputs *A* and *B* respectively.

With all the rates shown in 1 we extract the following ordinary differential equations that describe the change over time of the concentrations of *G*_1_, $G_{1}^{a}$, $G_{1}^{b}$, $G_{1}^{\mathrm{ab}}$, *X*, *A* and *B*:

(2) $$\begin{array}{l} dG_{1}/\mathrm{dt}=-k_{1}AG_{1}+K_{-1}G_{1}^{a}-k_{2}BG_{1}+k_{-2}G_{1}^{b}\\ dG_{1}^{a}/\mathrm{dt}=k_{1}AG_{1}-k_{-1}G_{1}^{a}-k_{3}BG_{1}^{a}+k_{-3}G_{1}^{\mathrm{ab}}\\ dG_{1}^{b}/\mathrm{dt}=k_{2}BG_{1}-k_{-2}G_{1}^{b}-k_{4}AG_{1}^{b}+k_{-4}G_{1}^{\mathrm{ab}}\\ dG_{1}^{\mathrm{ab}}/\mathrm{dt}=k_{3}BG_{1}^{a}-k_{-3}G_{1}^{\mathrm{ab}}+k_{4}AG_{1}^{b}-k_{-4}G_{1}^{\mathrm{ab}}\\ \mathrm{dX}/\mathrm{dt}=k_{5}G_{1}^{a}+k_{6}G_{1}^{b}+k_{7}G_{1}^{\mathrm{ab}}-k_{8}X\\ \mathrm{dA}/\mathrm{dt}=k_{11}-k_{1}AG_{1}-k_{4}AG_{1}^{b}-k_{9}A\\ \mathrm{dB}/\mathrm{dt}=k_{12}-k_{2}BG_{1}-k_{3}BG_{1}^{a}-k_{10}B \end{array}$$

All the parameter values used in 2 are taken from standard values in the literature [25-27]. The objective of this model is to provide as a generic model as possible, to abstract away from specific laboratory details (i.e. the utilisation of a concrete promoter type). Thus, similar kinetic parameters have the same value in order to prove the functioning of a complete *standardised* model. The values are as follows: *k*_1_ = *k*_2_ = *k*_3_ = *k*_4_ = 1 molecules^−1^hour^−1^; *k*_−1_ = *k*_−2_ = *k*_−3_ = *k*_−4_ = 50 hour^−1^; *k*_5_ = *k*_6_ = 500 hour^−1^; *k*_7_ = 700 hour^−1^; *k*_8_ = *k*_9_ = *k*_10_ = 0.1 hour^−1^; *k*_11_ = *k*_12_ = molecules hours^−1^. Notice that *k*_7_ is higher than *k*_5_and *k*_6_ in order to emphasise a stronger transcription rate when the two inputs are bound to their promoters at the same time [28].

**Figure 11:**
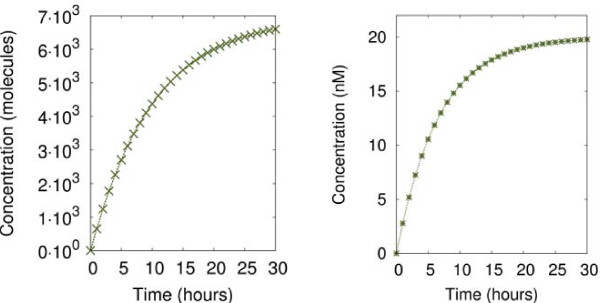
**Repressor concentrations over time.** Left graph: Repressor *X* concentration expressed over time, using the set of equations 2. Right graph: Repressor *X* concentration expressed over time in a simulation of equation 3.

With the initial conditions, which are *A* = *B* = 1000, and *G* = 1, we obtain Figure 11 (left), where we observe the concentration of *X* over time. In order to simplify the model we approximate the full deterministic model for the expression of *X* to a single equation. As two promoters control the expression of *X* (and *I*_1_), the promoters can be either additive or can interfere with each other [20]. Considering them to cooperate without interference, the equation for the rate of change of *X* over time is:

(3) $$\frac{\mathrm{dX}}{\mathrm{dt}}=\alpha_{X}\cdot \frac{{\left[A\right]}^{h_{1}}}{K_{d_{1}}+{\left[A\right]}^{h_{1}}}+\alpha_{X}\cdot \frac{{\left[B\right]}^{h_{1}}}{K_{d_{1}}+{\left[B\right]}^{h_{1}}}-\delta_{X}\cdot [X]$$

where [] denotes concentration. Figure 11 (right) show the behaviour of this equation over time. As we can see, both Figures 11 (left) and (right) show similar output curves over 30 hours (equivalent initial conditions as explained earlier). All the parameter values of equation 3 have been varied to agree with the behaviour of the full differential model. This validation allow us to express the rest of the system in 5 simplified equations, which are:

(4) $$\frac{dI_{1}}{\mathrm{dt}}=\alpha_{I_{1}}\cdot \frac{{\left[A\right]}^{h_{1}}}{K_{d_{2}}+{\left[A\right]}^{h_{1}}}+\alpha_{I_{1}}\cdot \frac{{\left[B\right]}^{h_{1}}}{K_{d_{2}}+{\left[B\right]}^{h_{1}}}-\delta_{I_{1}}\cdot [I_{1}]$$

(5) $$\frac{dI_{2}}{\mathrm{dt}}=\alpha_{I_{2}}\cdot \frac{1}{1{+(\frac{\left[X\right]}{\beta_{I_{2}}})}^{h_{2}}}-\delta_{I_{2}}\cdot [I_{2}]$$

(6) $$\frac{dR_{1}}{\mathrm{dt}}=\alpha_{R_{1}}\cdot \frac{1}{1+{\left(\frac{\left[\max([R_{2}]-[I_{2}],0)\right]}{\beta_{R_{1}}}\right)}^{h_{2}}}-\delta_{R_{1}}\cdot [R_{1}]$$

(7) $$\frac{dR_{2}}{\mathrm{dt}}=\alpha_{R_{2}}\cdot \frac{1}{1+{\left(\frac{\left[\max([R_{1}]-[I_{1}],0)\right]}{\beta_{R_{2}}}\right)}^{h_{2}}}-\delta_{R_{2}}\cdot [R_{2}]$$

(8) $$\frac{\mathrm{dOut}}{\mathrm{dt}}=\alpha_{\mathrm{Out}}\cdot \frac{1}{1+{\left(\frac{\left[\max(R_{2}-I_{2},0)\right]}{\beta_{\mathrm{Out}}}\right)}^{h_{2}}}-\delta_{\mathrm{Out}}\cdot [\mathrm{Out}]$$

where *α*denotes a synthesis rate, *K* represents the dissociation constants, *h* the Hill coefficients, *δ* the protein decay or degradation rate and *β*denotes repression coefficients. It is important to notice that the sequestrated repressor complexes, *C*_1_ and *C*_2_, are represented by *max*([*R*_1_]−[*I*_1_],0) and *max*([*R*_2_]−[*I*_2_],0) which are the direct subtraction of the repressor by the inducer (or 0 if a negative result is obtained).

The values of the parameters are chosen to make Figure 11 (right) match Figure 11 (left) according to standard values [26,29]. As before, similar parameters have the same value in order to make the *in-silico* study as general as possible. Thus, $\alpha_{I_{1}}$ = *α*_*X*_ = 3.0 nM hour^−1^; $\alpha_{I_{2}}$ = $\alpha_{R_{1}}$ = $\alpha_{R_{2}}$ = *α*_*Out*_ = 4.0 nM hour^−1^; $K_{d_{1}}$ = $K_{d_{2}}$ = 0.5 nM; $\beta_{I_{2}}$ = $\beta_{R_{1}}$ = $\beta_{R_{2}}$ = *β*_*Out*_ = 0.04 nM; *δ*_*i*_ = 0.15 hour^−1^; *h*_1_ = 1; *h*_2_ = 2. The initial conditions are: *A*= *B* = 0.5 nM (again, both inputs set to a logic *1*).

All simulations are performed with our own software coded in *Python*. Figures 2, 3, 8, 9 and 11 are obtained by using a deterministic approach with the previous ODE (Ordinary Differential Equation) model. For Figure 4 we added noise to inputs, as explained in the Results and discussion section, without changing the ODEs. Figure 5 is obtained by adding noise to the full model (all species that change over time). For that purpose, the ODE model is altered by adding Gaussian noise at every integration step to the previous concentration (building a SDE, Stochastic Differential Equation model). In the spatial studies (Figures 6, 7 and 10) the inputs are fixed in their specific surface areas and the rest of the species are subject to low stochasticity (in this case, however, it is the collective behaviour that matters, not individual).

For spatial studies we use the physics library *Pymunk* (wrapper for the physics library *Chipmunk*) to design and control the cells as rigid bodies with growth in an agent-based paradigm. The ODE model of the system is *placed* inside the cells so there are as many copies of the genetic circuit as cells in a given time (the circuit with all parameter values is copied from mother to daughter when the cell divides). In order to control the doubling time of the cells we can let the circuit run for as many integration steps as we may need before the cell divides. For visualisation purposes, the circuit runs during 12 or 24 hours in a cell life-time (making that time the doubling time) depending on the set-up (see Results and discussion section).

## Competing interests

The authors declare that they have no competing interests.

## Authors’ contributions

AG-M conceived the study, designed the circuit and performed the experiments. MA supervised the work. Both authors participated in writing the manuscript, and both read and approved the final manuscript.
